# Polo kinase regulates the localization and activity of the chromosomal passenger complex in meiosis and mitosis in *Drosophila melanogaster*

**DOI:** 10.1098/rsob.140162

**Published:** 2014-11-05

**Authors:** Mar Carmena, Miguel Ortiz Lombardia, Hiromi Ogawa, William C. Earnshaw

**Affiliations:** 1 The Wellcome Trust Centre for Cell Biology, Institute of Cell Biology, University of Edinburgh, Edinburgh EH9 3JR, UK; 2 Centre National de la Recherche Scientifique, Aix-Marseille Université, CNRS UMR 7257, AFMB, 163 Avenue de Luminy, 13288 Marseille, France

**Keywords:** chromosomal passenger complex, Aurora kinases, Polo-like kinases, mitosis, meiosis, *Drosophila*

## Abstract

Cell cycle progression is regulated by members of the cyclin-dependent kinase (CDK), Polo and Aurora families of protein kinases. The levels of expression and localization of the key regulatory kinases are themselves subject to very tight control. There is increasing evidence that crosstalk between the mitotic kinases provides for an additional level of regulation. We have previously shown that Aurora B activates Polo kinase at the centromere in mitosis, and that the interaction between Polo and the chromosomal passenger complex (CPC) component INCENP is essential in this activation. In this report, we show that Polo kinase is required for the correct localization and activity of the CPC in meiosis and mitosis. Study of the phenotype of different *polo* allele combinations compared to the effect of chemical inhibition revealed significant differences in the localization and activity of the CPC in diploid tissues. Our results shed new light on the mechanisms that control the activity of Aurora B in meiosis and mitosis.

## 2. Introduction

Cell cycle progression is regulated by reversible phosphorylation [1]. Protein kinases and phosphatases control the correct levels of phosphorylation of key substrates to ensure smooth progression of the cell cycle [1,2]. Frequently, these substrates are themselves kinases or phosphatases that form part of complex regulatory networks involving multiple feedback loops [1]. A group of highly conserved protein kinases is responsible for the overall control of these regulatory networks. This group includes the families of cyclin-dependent kinases (CDKs) [3], Aurora kinases [4,5] and Polo-like kinases (Plks) [6]. Misexpression of these protein kinases is linked to aneuploidy and carcinogenesis, making them very attractive targets for the development of new anti-cancer therapies [7–10]. The levels and activity of the master regulatory kinases must therefore be very tightly regulated.

Regulation of the mitotic kinases occurs at multiple levels including modulation of their expression, proteolysis and targeting to different subcellular locations. Additionally, their enzymatic activity is regulated by specific cofactors and by the level of phosphorylation of activation segments—either by autophosphorylation or by the action of other kinases and phosphatases (for reviews, see [5,6,11]). There is increasing evidence of crossregulation among CDKs, Polo-like and Aurora kinases. Polo-like kinase 1 (Plk1) modulates CDK1 activity through phosphorylation of several CDK1 regulators: promoting accumulation in the nucleus of Cdc25C [12], the degradation of Wee1 [13] and inhibition of Myt1 [14]. CDK1 acts as a priming kinase regulating the docking of Plk1 to its substrates (i.e. Bub1 [15] and BubR1 [16]). Aurora A and its interactor Bora are responsible for the activation of Plk1 at the centrosome at the G2–M transition [17,18] and in human cells contribute to its activation later in mitosis [19]. CDK1 phosphorylation of Bora enhances binding to Plk1 [20,21] and therefore promotes activation by Aurora A. Conversely, Plk1 regulates degradation of both Bora [18,21] and Aurora A [22]. We recently demonstrated that Aurora B is the kinase responsible for the activation of Polo kinase at the centromere [23] in mitosis, and that the chromosomal passenger complex (CPC) component INCENP is essential for this activation. Importantly, we showed that this regulatory mechanism plays an essential role in diploid tissues *in vivo* and not only in cultured aneuploid cell lines.

In this study, we have analysed the regulation of the CPC by Polo kinase in meiosis and mitosis in *Drosophila*. Using different combinations of *polo* mutant alleles, we show that Polo kinase is required for the correct localization and activity of the CPC at all stages of male meiosis as well as in larval neuroblast mitoses. This analysis reveals differences in the regulation of the centromeric localization of the CPC by Polo kinase between the two meiotic divisions. In addition, we show that chemical inhibition of Polo kinase activity in neuroblasts phenocopies the CPC defects in localization and activity observed in *polo* mutants. Interestingly, analysis of the neuroblast mitoses revealed significant differences between the phenotypes resulting from the depletion of Polo kinase protein in comparison with the inhibition of its kinase activity.

## 3. Material and methods

### 3.1. *Drosophila* strains

Fly strains were grown at 25°C in standard *Drosophila* medium. The following stocks were used: Canton-S. *polo^1^/TM6C. w; polo^9^/TM6C. w; polo^10^/TM6C*. Immunostaining of testes and third instar larval neuroblasts was performed as described previously [24]. For drug treatment, larval neuroblasts were dissected and treated with either dimethylsulfoxide (DMSO) or 100 nM BI 2536 for 2 h before being processed for immunostaining as described previously [23].

### 3.2. Antibodies

Primary antibodies and dilutions for immunofluorescence analysis were as follows: mouse monoclonal B512 anti-αTubulin (SIGMA, 1 : 2000); rabbit polyclonal anti-INCENP Rb-801, Rb-803 [25], 1 : 500; mouse monoclonal anti- Plk1^T210Ph^ (Abcam ab39068, 1 : 100) and rabbit polyclonal anti-Histone3^Ser10Ph^ (Upstate, 1 : 500). Secondary antibodies were obtained from Jackson Immunoresearch.

### 3.3. *Drosophila* cell culture, drug treatment and immunofluorescence

*Drosophila* cell lines were grown in Express-Five medium (GIBCO). The AC5-Polo-GFP cell line was described previously [26]. Cells exponentially growing were seeded on Con-A treated coverslips and treated with either DMSO or 100 nM BI 2536 for 2 h before being processed for immunostaining as described previously [23,25]. Imaging was performed using an Olympus IX-71 microscope controlled by Delta Vision SoftWorx (Applied Precision, Issequa, WA, USA). Image stacks were deconvolved, quick-projected, and saved as tiff images to be processed using Adobe PhotoShop.

Signal intensities were measured using the softWoRx Data Inspector tool; average background was subtracted; data were plotted using Prism software.

## 4. Results

### 4.1. Polo kinase is required for the correct localization of the chromosomal passenger complex in meiosis I

**Figure 1.**
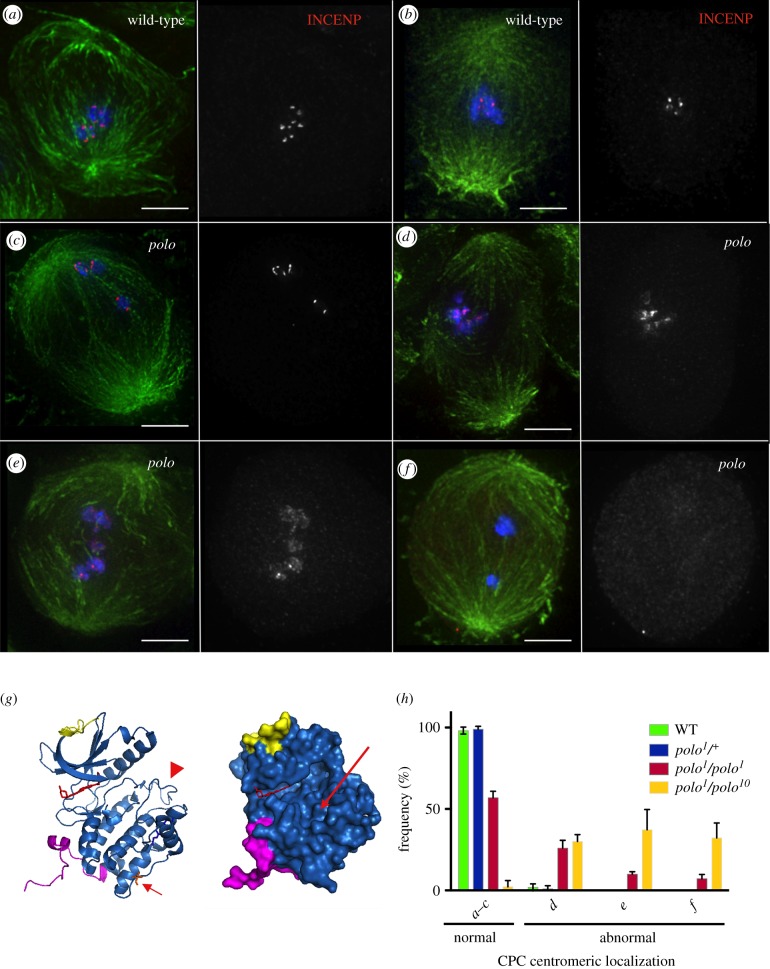
The CPC is mislocalized in *polo* mutant meiosis I. In wild-type spermatocytes the CPC concentrates at the centromeres in prometaphase I (*a*) and metaphase I (*b*). In all *polo* mutant combinations, we observe differences in the CPC localization in spermatocytes. The different phenotypic categories observed are shown in (*c*–*f*). These phenotypes range from normal localization (*c*), slight dispersion to surrounding heterochromatin (*d*), diffuse localization all over the chromatin (*e*) or reduction/absence of CPC signal (*f*). Green, α-tubulin; red, INCENP; blue, DNA; scale bars, 1 µm. (*g*) Predicted effect of the *polo^1^* mutation on kinase function. Left, structure of human Plk1 kinase domain (PDB code 2OWB). Arrowhead points to the T-loop; arrow points to the residue mutated in *polo^1^* (valine 242). Right, surface view of the kinase domain. Long arrow points to substrate-binding groove, formed by subdomains VIII and IX. (*h*) Quantification of the percentages of each phenotypic category (as shown in *c*–*f*) in the different *polo* mutant allelic combinations studied.

In order to investigate the role of Polo kinase in the regulation of the CPC in meiosis, we decided to study the distribution of the CPC component INCENP in *Drosophila* spermatogenesis in different *polo* mutant allelic combinations. In prometaphase and in metaphase I wild-type spermatocytes, the CPC concentrates at centromeres (figure 1*a,b*). The centromeric levels of the complex in metaphase I appear considerably reduced compared to prometaphase I.

In *polo* mutants, we observe the following patterns of CPC localization (figure 1*c–f*): INCENP is found either normally concentrated at centromeres (figure 1*c*), slightly dispersed on the region surrounding the centromeres (figure 1*d*), completely dispersed all over the chromatin (figure 1*e*) or highly reduced/absent from chromatin (figure 1*f*). These phenotypic categories were quantified in different allelic combinations of *polo* mutations that result in a decrease of either the levels or the activity of the kinase.

The original *polo^1^* mutation is a point mutation resulting in a substitution of valine 242 in the kinase domain by glutamic acid (figure 1*g*, left panel, arrow). Mapping this mutation in the structure of human PLK1 kinase domain (PDB code 2OWB; figure 1*g*, left, arrow) shows that this substitution would disrupt hydrophobic contacts with the first helix of kinase subdomain XI. We predict that such disruption may alter the relative positions of subdomains X, where valine 242 sits, and IX, which in turn would affect the substrate-binding groove, formed by subdomains VIII and IX (figure 1*g*, right, arrow). Subdomain VIII is also important for the stability of the kinase domain: first, via a nearly invariant ion pair with subdomain XI, secondly, by its direct interaction with the activation loop (figure 1*g*, left, arrowhead). As a result of this substitution, the Polo^1^ mutant kinase is predicted to have reduced enzymatic activity and be compromised in its substrate recognition. The expression levels of the mutant kinase are similar to wild-type [27]. By contrast, the *polo^9^* and *polo^10^* mutations are P-element insertions in the upstream regulatory region of the gene that result in a dramatic reduction of Polo expression levels [28].

Our analysis shows that the reduction in kinase activity caused by the *polo^1^* mutation results in CPC localization defects (figure 1*h*). However in over 50% of homozygous *polo^1^* mutant spermatocytes, the CPC appears normally localized at centromeres (in some instances even when bivalents are missegregating, figure 1*c*). A further reduction of protein levels in the *polo^1^/polo^10^* (and *polo^1^/polo^9^*, data not shown) results in a significant increase in the percentage of spermatocytes showing abnormal CPC localization (figure 1*h*).

**Figure 2.**
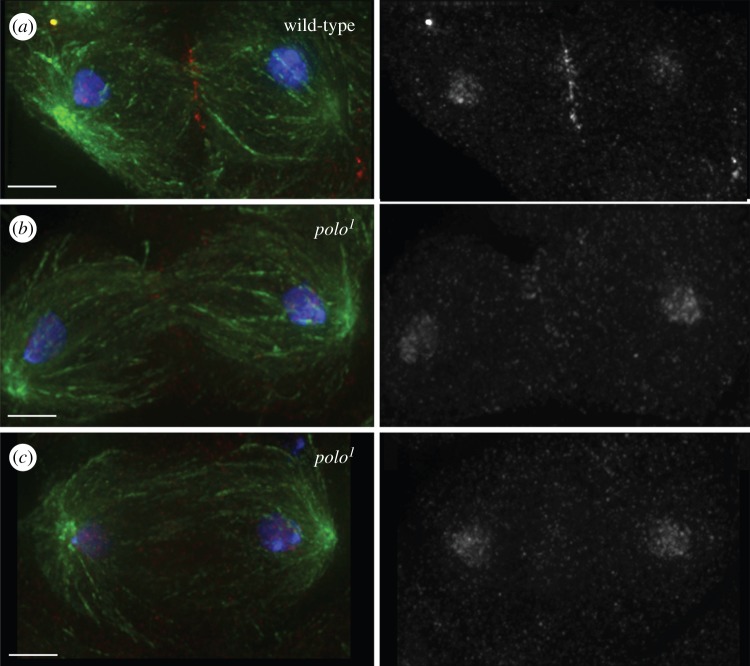
The CPC is mislocalized in late meiosis I in *polo^1^* mutants. (*a*) Wild-type telophase I spermatocyte showing correct transfer of the CPC to the central spindle. (*b,c*) *polo^1^*/*polo^1^*mutant spermatocyte showing defective CPC transfer to the midzone in the presence (*b*) or the absence (*c*) of normal central spindle. Green, tubulin; red, INCENP; blue, DNA; scale bars, 5 µm.

A considerable proportion (31%) of homozygous *polo^1^* mutant cysts undergoing meiosis I shows defects in late anaphase and cytokinesis [24]. These include defects in the formation of both the spindle midzone and the contractile ring. As the CPC is critically involved in the regulation of events in late mitosis and cytokinesis, we wanted to find out if the phenotypes found in *polo^1^* mutants are at least in part a consequence of a disruption of CPC localization or function. The dynamic localization of the CPC shows important differences in meiosis: at the metaphase I to anaphase I transition part of the CPC transfers to the central spindle as it does in mitosis, but a subset of the CPC remains associated with chromatin ([29] and figure 2). Analysis of late stages of meiosis I in *polo^1^* mutants revealed that the CPC does not transfer to microtubules correctly at the metaphase to anaphase transition (figure 2*b*,*c*). This defective CPC localization is independent of the degree of disruption of the spindle midzone, as visualized by staining for microtubules (cf. figure 2*b*,*c*).

### 4.2. Polo kinase function is required for the correct localization of the chromosomal passenger complex in meiosis II

**Figure 3.**
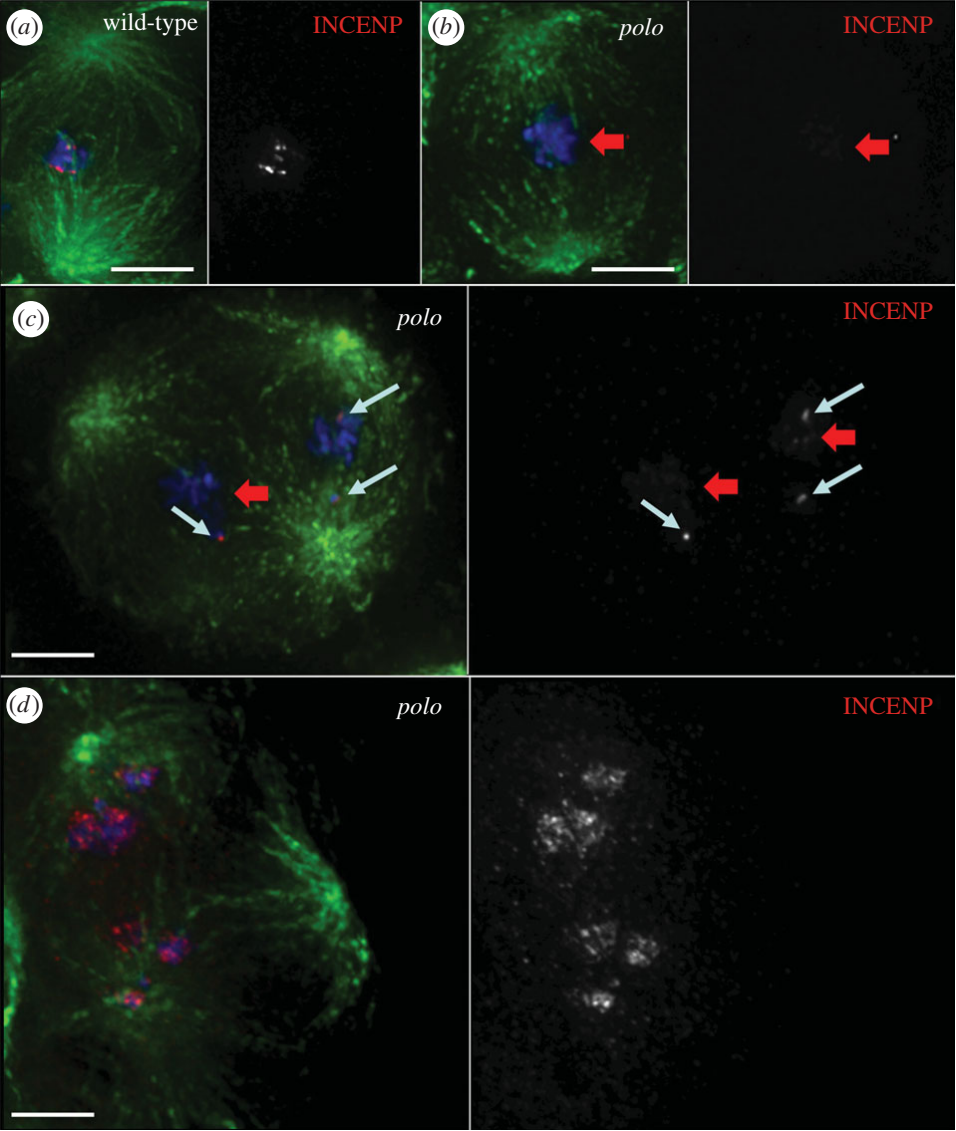
Tension-dependent CPC mislocalization in *polo* mutant meiosis II. In wild-type spermatocytes, the CPC concentrates at the centromeres in metaphase II (*a*). In *polo* mutant spermatocytes, we observe defects in CPC localization (*b*–*d*). In *polo^1^*/*polo^1^* metaphase II spermatocytes the CPC is frequently absent from aligned chromosomes (*b,c*, red arrows) while it is normally concentrated at centromeres of unaligned chromosomes (*c*, white arrows). (*d*) In *polo^1^*/*polo^10^* spermatocytes, the predominant phenotype is a diffuse localization of the CPC all over the chromatin. Green, α-tubulin; red, INCENP; blue, DNA; scale bars, 5 µm.

A small percentage of homozygous *polo^1^* mutant spermatocytes complete meiosis I and proceed to meiosis II. Spermatocytes that fail cytokinesis in meiosis I form multipolar spindles in meiosis II (figure 3*c*,*d*). In wild-type spermatocytes, the localization of the CPC in prometaphase and metaphase II resembles that in the first meiotic division, with the CPC concentrating at centromeres (figure 3*a*). As spermatocytes within each cyst undergo meiosis in a slightly asynchronous way, we are able to observe consecutive stages of the meiotic division side by side in single cysts. In *polo^1^* mutant cysts in prometaphase II, the CPC localizes properly to the centromeres of unaligned chromosomes (figure 3*c*, white arrows), but it is virtually undetectable on chromosomes aligned at the equatorial plate (figure 3*c*, red arrows). In these cells, once the spermatocytes reach metaphase II, the CPC is virtually undetectable on chromosomes (figure 3*b*). We conclude that Polo kinase activity is not required for the initial targeting and concentration of the CPC to the centromeres in meiosis II, but it is required for the stable localization of the complex, possibly in a tension-dependent manner. In some instances, we also observe the CPC dispersed all over the chromatin in prometaphase and metaphase II, similarly to what occurs in meiosis I (figure 3*d*).

### 4.3. Polo kinase is required for the correct localization and activity of the chromosomal passenger complex in *Drosophila* larval neuroblast mitoses

**Figure 4.**
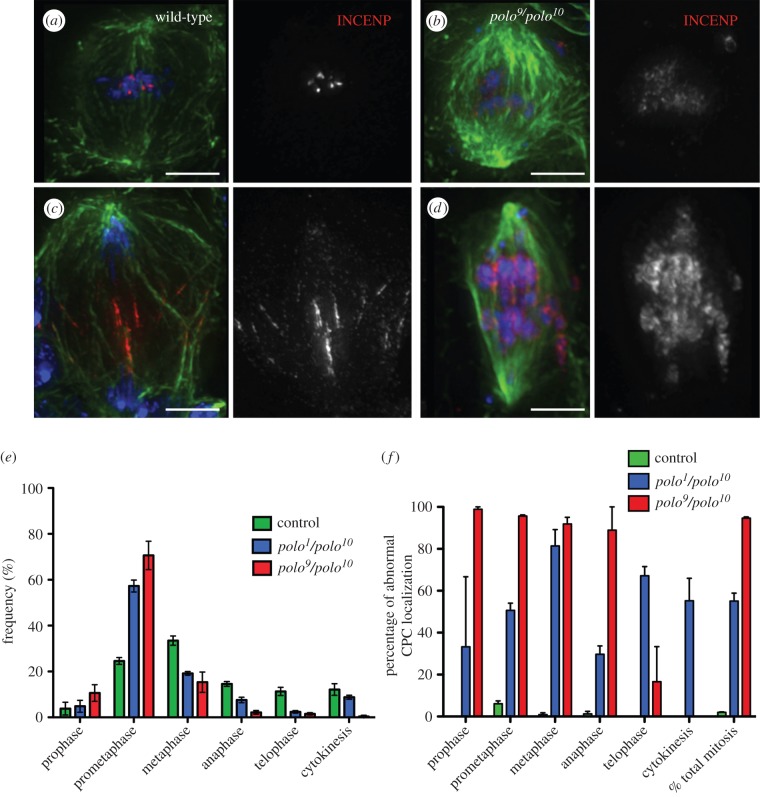
Localization of the CPC is abnormal in *polo* mutant neuroblast mitoses. In wild-type third instar larval neuroblasts, the CPC concentrates at the centromeres in metaphase (*a*) and transfers to the spindle microtubules and cortex in anaphase (*c*). In *polo^9^/polo^10^* mutants, INCENP is dispersed on the chromatin in mitosis (*b,d*). Green, α-tubulin; red, INCENP; blue, DNA; scale bars, 5 µm. (*e*) Distribution of cells in the different stages of mitosis and cytokinesis. (*f*) Frequency of cells showing abnormal CPC localization in different stages of mitosis and cytokinesis (error bars = standard deviation from triplicate experiments).

In order to analyse the consequences of further depletion of the Polo kinase protein levels in CPC function, we studied mitosis in *polo^9^/polo^10^*mutants. Flies carrying this allelic combination are late larval lethal. Thus, it is not possible to study meiotic phenotypes, since the testes are not yet mature. Instead we studied the mitotic phenotypes in third instar larval neuroblasts in *polo* mutants. Wild-type neuroblasts are actively dividing, with a mitotic index of 1.8 ± 0.21% (*n* = 1000 per experiment). The mitotic index is significantly higher in *polo^9^/polo^10^*and *polo^1^/polo^10^* neuroblasts (26.7 ± 1.9% and 8.6 ± 0.26%, respectively, *n* = 1000 per experiment). In most mitotic figures analysed, the CPC shows an abnormal pattern of localization and is dispersed all over the chromatin (figure 4*b,d*; electronic supplementary material, figure S1).

Analysis of the distribution of mitotic phases shows a significant increase in the proportion of neuroblasts in prometaphase. This is accompanied by a decrease in the frequency of cells in later mitotic stages (up to 10 times less from 38% in wild-type to 3.9% in *polo^9^/polo^10^*, figure 4*e*). This distribution is a consequence of the characteristic prometaphase delay in *polo* mutants. Defects in CPC localization are observed in all stages of mitosis with high frequency, especially in *polo^9^/polo^10^* where 95% of cells in mitosis show abnormal INCENP distribution (figure 4*f*). Compared to in *polo^9^/polo^10^*, in *polo^1^/polo^10^* mutants there is a slightly higher frequency of neuroblasts in anaphase (figure 4*e*). In these cells, the CPC remains associated with segregating chromatids and does not transfer normally to the central spindle microtubules (electronic supplementary material, figure S1*d*), similarly to what we observe in meiosis I. These abnormal mitoses exhibit totally depleted or barely undetectable levels of Polo^Ph-Thr182^, the active form of the kinase (electronic supplementary material, figure S2).

**Figure 5.**
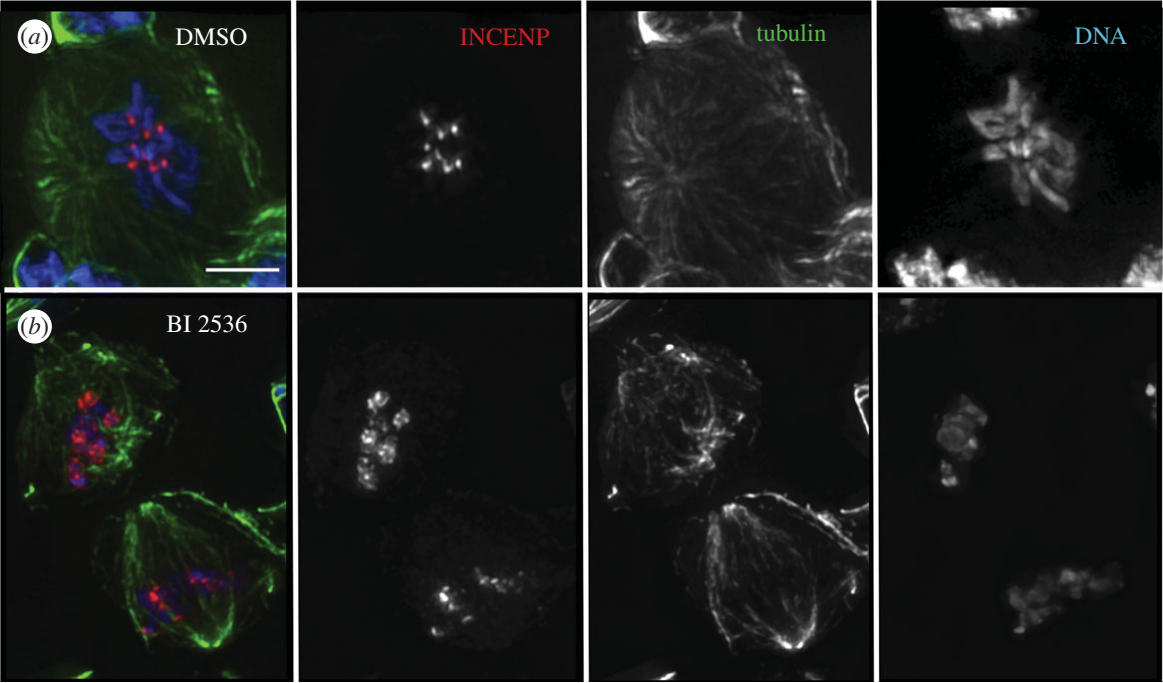
Treatment with Plk1 inhibitor BI 2536 phenocopies *polo* mutant phenotype in neuroblast mitoses. (*a*) Control (DMSO-treated) third instar larval neuroblasts show normal localization of the CPC, whereas BI 2536-treated ones (*b*) show CPC mislocalization phenotypes similar to those observed in *polo* mutants. Green, α-tubulin; red, INCENP; blue, DNA; scale bars, 5 µm.

**Figure 6.**
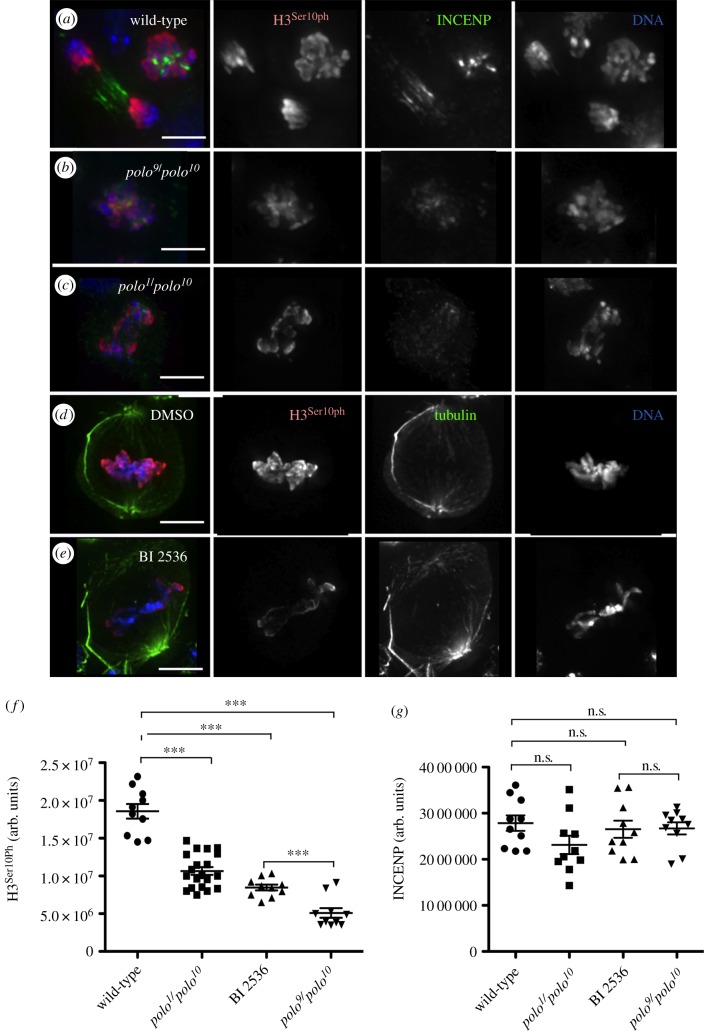
Aurora B activity is reduced in *polo* mutant and BI 2536-treated neuroblast mitoses. (*a*–*c*) Levels of phosphorylation of Histone3-Ser10 in neuroblast mitoses. (*a*) wild-type, (*b*) *polo^9^/polo^10^*mutants, (*c*) *polo^1^/polo^10^* mutants. Green, INCENP; red, PhosphoHistone3-Ser10; blue, DNA. (*d*–*e*) Levels of phosphorylation of Histone3-Ser10 in DMSO (*d*) and BI 2536-treated (*e*) neuroblasts. Green, tubulin; red, PhosphoHistone3 Ser10; blue, DNA; scale bars, 5 µm. (*f*–*g*) Quantification of the levels of Histone 3-Ser10 phosphorylation (*f*) and INCENP (*g*) in *polo* mutant combinations and BI 2536-treated neuroblasts. In (*f*), *t*-test comparing wild-type with each of three experiments shows differences are significant (***p < 0.0001); difference between BI 2536-treated and *polo^9^/polo^10^* is also significant (***p < 0.0003).

We next questioned whether the observed defects in CPC localization had an impact on the activity of Aurora B kinase. We monitored this by quantification of the levels of phosphorylation of the Aurora B substrate Histone3 Serine10—H3^Ser10Ph^ (and H3^Ser28Ph^, data not shown). In order to assess the difference between the effects of depleting Polo protein levels in *polo* mutants versus those resulting from inhibition of its kinase activity (in which the protein is still present), we compared the levels of H3^Ser10Ph^ in wild-type neuroblasts treated with the Plk1 inhibitor BI 2536 with those observed in neuroblasts from various combinations of *polo* mutants (figures 5 and 6; electronic supplementary material, figures S3 and S4). BI 2536 treatment of wild-type neuroblasts results in an elevated mitotic index compared to wild-type (18 ± 1.2%; *n* = 1000 per experiment). Drug treatment also phenocopies the CPC mislocalization phenotypes observed in *polo* mutants (figure 5). Thus, the phenotype observed after treatment with inhibitor has a much higher penetrance than that observed in *polo^1^/polo^1^* neuroblasts (data not shown). This is most probably explained by the residual enzymatic activity in the Polo^1^ mutant kinase.

Although in all experimental conditions we observe a reduction of H3^Ser10Ph^ levels compared to the levels of the wild-type (figure 6*f*), the biggest reduction is found in the *polo^9^/polo^10^*mutant (figure 6*b*). Interestingly, the level of phosphorylation in *polo^9^/polo^10^* mutant neuroblasts is significantly lower than that in the BI 2536-treated neuroblasts (*p* = 0.0003, figure 6*f*; electronic supplementary material, figure S4). Thus, as far as the activity of Aurora B is concerned, the reduction of Polo protein levels in the mutant neuroblasts has a stronger effect than inhibition of Polo kinase activity.

### 4.4. Polo does not require its own kinase activity for localization to centromeres in *Drosophila*

**Figure 7.**
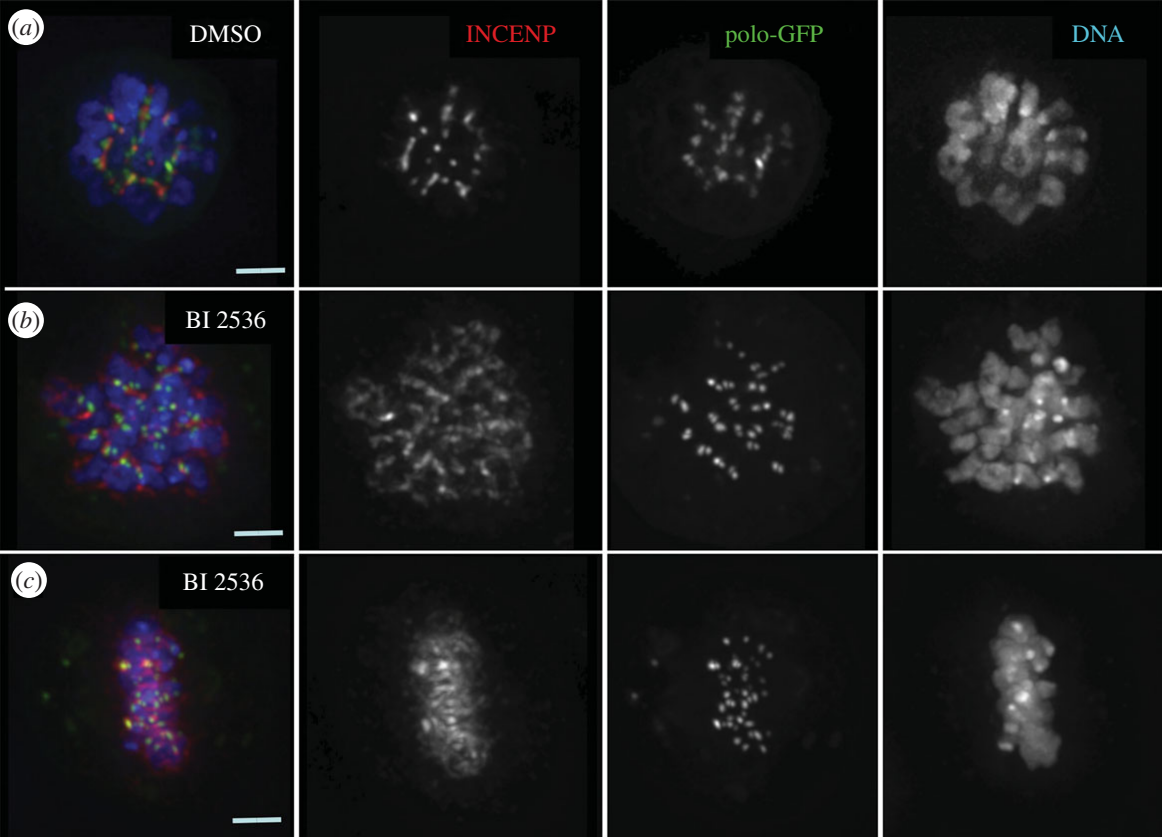
Polo kinase does not require its own kinase activity for kinetochore localization. D-Mel2 cells stably expressing Polo-GFP were treated with either DMSO (*a*) or the Plk1 inhibitor BI 2536 (*b*,*c*). Red, INCENP; green, Polo-GFP; blue, DNA; scale bars, 5 µm.

In order to understand better the different effects observed as a consequence of either lowering the levels of Polo or inhibiting its kinase activity, we analysed the localization of the kinase in DMel2 cultured cells treated with the BI 2536 inhibitor. Similarly to what we observed in dividing neuroblasts, the distribution of the CPC is abnormal in inhibitor-treated cells (figure 7). Interestingly, and in contrast to what has been described in human cells, Polo kinase localizes normally to the kinetochore in DMel2 cells (figure 7*b,c*).

## 5. Discussion

Members of the CDK, Polo and Aurora kinase families are frequently involved in the regulation of the same cell cycle events, and even act upon the same substrates. Coordination of the activities of these highly conserved kinases is therefore essential for the smooth progression of cell division. One way that the cell accomplishes this coordination is by making the activity of one key regulatory kinase dependent on another one. For example, activation of CDK1 is accomplished by a bistable system that depends on feedback loops that both activate CDC25 and inactivate WEE1 (reviewed in [30]). Plk1 is responsible for the activation of this bistable switch [31]. In turn, CDK1 is frequently the priming kinase that allows binding of Plk1 to its substrates in mitosis through its polo-box domain [32]. In addition, the activation of Plk1 at the G2–M transition depends on Aurora A-Bora [17,18]. In a recent study, we reported that the activation of Polo kinase at the centromere depends on Aurora B kinase activity and is mediated by the CPC component INCENP [23].

Here, we show that the localization and activity of the CPC depends on Polo kinase in male meiosis and neuroblast mitosis in *Drosophila*. A similar dependence was reported previously in tissue culture cells [33]. Here for the first time, we were able to study the regulation of the CPC by Polo kinase in all phases of male meiosis. This was made possible thanks to the use of the weaker *polo^1^* allele (either homozygous or in combination with stronger *polo* alleles). A recent study examining the effect of chemical inhibition of Polo kinase in *Drosophila* spermatocytes showed a much stronger phenotype than that of *polo^1^* mutants [34]. In that study, BI 2536-treated spermatocytes were blocked in a prometaphase-like state with condensed bivalents that did not divide. Our analysis showed that Polo is essential not only for the correct centromeric localization of the CPC in both meiotic divisions but also for transfer of the CPC to the central spindle in anaphase. Similar phenotypes were also observed in neuroblast mitoses (figure 4; electronic supplementary material, figure S1).

Analysis of different combinations of *polo* alleles allowed us to compare the effect of depletion of Polo protein levels versus inhibition of its kinase activity on CPC localization and activity. Quantification of the different CPC localization phenotypes in meiosis I revealed that partial inhibition of the kinase activity in *polo^1^* homozygotes resulted in a majority of spermatocytes showing mild (figure 1*d*) or no defects (figure 1*c*). Further decreasing the level of Polo kinase (*polo^1^/polo^10^*) resulted in a significant increase in the proportion of cells showing the most extreme CPC mislocalization phenotype, with the complex spread all over the chromatin. It is tempting to predict that if we could analyse the CPC meiotic localization in *polo^9^* and *polo^10^* mutants we would find an even higher proportion of this extreme phenotype. This was indeed the case when we analysed the mitotic phenotype in *polo^9^*/*polo^10^* neuroblasts (see below).

Contrary to what was described for BI 2536-treated human cells [35], where cells are delayed in prophase with monopolar spindles, inhibitor-treated and *polo* mutant *Drosophila* neuroblasts form bipolar spindles but exhibit a prometaphase delay. This is true for all allelic combinations studied and also for BI 2536-treated neuroblasts (figure 5 and data not shown).

Altogether these results indicate a significant difference between the kinase inhibition and depletion phenotypes. Additionally, in Polo kinase depleted (*polo^9^*/*polo^10^*) neuroblasts 95% of the mitotic cells show the CPC dispersed all over the chromatin, a much higher proportion than is found in BI 2536-treated neuroblasts. As we have shown that inactive Polo kinase retains the ability to target to the centromere in *Drosophila* (figure 7), we propose that the mutant kinase could also retain some capacity to dock the CPC, thereby resulting in a relatively more stable centromeric localization of the complex.

Cytokinesis requires both Polo and Aurora B kinase function [6,11,36,37]. Plk1 is required for the regulation of cytokinesis in all species studied [11,24,38,39]. It has been proposed that Plk1 inhibitory phosphorylation of PRC1 prevents premature midzone assembly [40]. Although Plk1 is known to act in part through the activation of the GTPase RhoA at the actomyosin ring [39,41,42], the roles and substrates of Plk1 in cytokinesis are not completely characterized. The CPC regulates abscission, the last stage of cytokinesis [43–45]. Our results show that the CPC does not transfer to the central spindle normally in anaphase in both mitosis and meiosis of *polo* mutants. In flies and human cells, subito/MKLP2 binds to Aurora B and INCENP and is required for the correct localization of the CPC to the central spindle microtubules in anaphase [46,47]. Plk1 binds and phosphorylates MKLP2, negatively regulating its microtubule bundling activity. However, phosphorylation by Plk1 is not required for the localization of MKLP2 to the spindle midzone [46–48]. Our results indicate that if the CPC depends on Polo for its correct positioning at the spindle midzone, this must occur through an alternative pathway. Additionally, our results suggest that at least part of the Polo requirement in cytokinesis could be explained by its role in CPC localization and function. However, at this point we cannot exclude that the CPC mislocalization phenotype might be a secondary consequence of central spindle defects in the *polo* mutants.

The intricate web of interactions between CDK1, Polo and the CPC is critical throughout mitosis. Here, we have shown that not only do these kinases regulate one another by adjusting their activity levels, but they also have a role in regulating mitotic progression by ensuring that the kinases are active in the right place at the right time.

## Supplementary Material

Suplemental Figure Legends

## Supplementary Material

Supplemental Figure 1

## Supplementary Material

Supplemental Figure 2

## Supplementary Material

Supplemental Figure 3

## Supplementary Material

Supplemental Figure 4
