# Supplementary material for: Polo kinase regulates the localization and activity of the chromosomal passenger complex in meiosis and mitosis in Drosophila melanogaster
Source: Open Biol. 2014 Nov 5;4(11):140162. doi: 10.1098/rsob.140162 (PMC4248065; doi:10.1098/rsob.140162)
Supplement: Suplemental Figure Legends [file rsob140162supp1.docx]

**SUPPLEMENTAL FIGURES**

**Supplemental Figure 1. Localization of the CPC is abnormal in *polo* mutant neuroblast mitoses.**

In wild type third instar larval neuroblasts the CPC concentrates at the centromeres in metaphase (A) and transfers to the spindle microtubules and cortex in anaphase (C). In *polo^1^/polo^10^* mutants INCENP is dispersed on the chromatin in mitosis (B and D).

**Supplemental Figure 2. Levels of active Polo kinase are barely detectable in *polo^9^/polo^10^* mutants.**

Immunostaining of third instar larval neuroblasts **(**Green: Polo^Ph-Thr182^ ; red: INCENP) in A) wild type and B-C) *polo^9^/polo^10^* mutants.

**Supplemental Figure 3. Aurora B activity is reduced in *polo^1^/polo^10^* mutant neuroblast mitoses.**

Levels of phosphorylation of Histone3 Ser10 in (A) wild-type and (B-C) *polo^1^/polo^10^* mutants. (Green: INCENP; red: PhosphoHistone3 Ser10; blue: DNA).

**Supplemental Figure 4. Aurora B activity is reduced in BI 2536-treated neuroblast mitoses.**

Levels of phosphorylation of Histone3 Ser10 in (A, C, E) wild-type and (B, D, F) BI 2536-treated neuroblasts. (Green: tubulin; red: PhosphoHistone3 Ser10; blue: DNA).
