# Supplementary figures and images for: Polo kinase regulates the localization and activity of the chromosomal passenger complex in meiosis and mitosis in Drosophila melanogaster
Source: Open Biol. 2014 Nov 5;4(11):140162. doi: 10.1098/rsob.140162 (PMC4248065; doi:10.1098/rsob.140162)

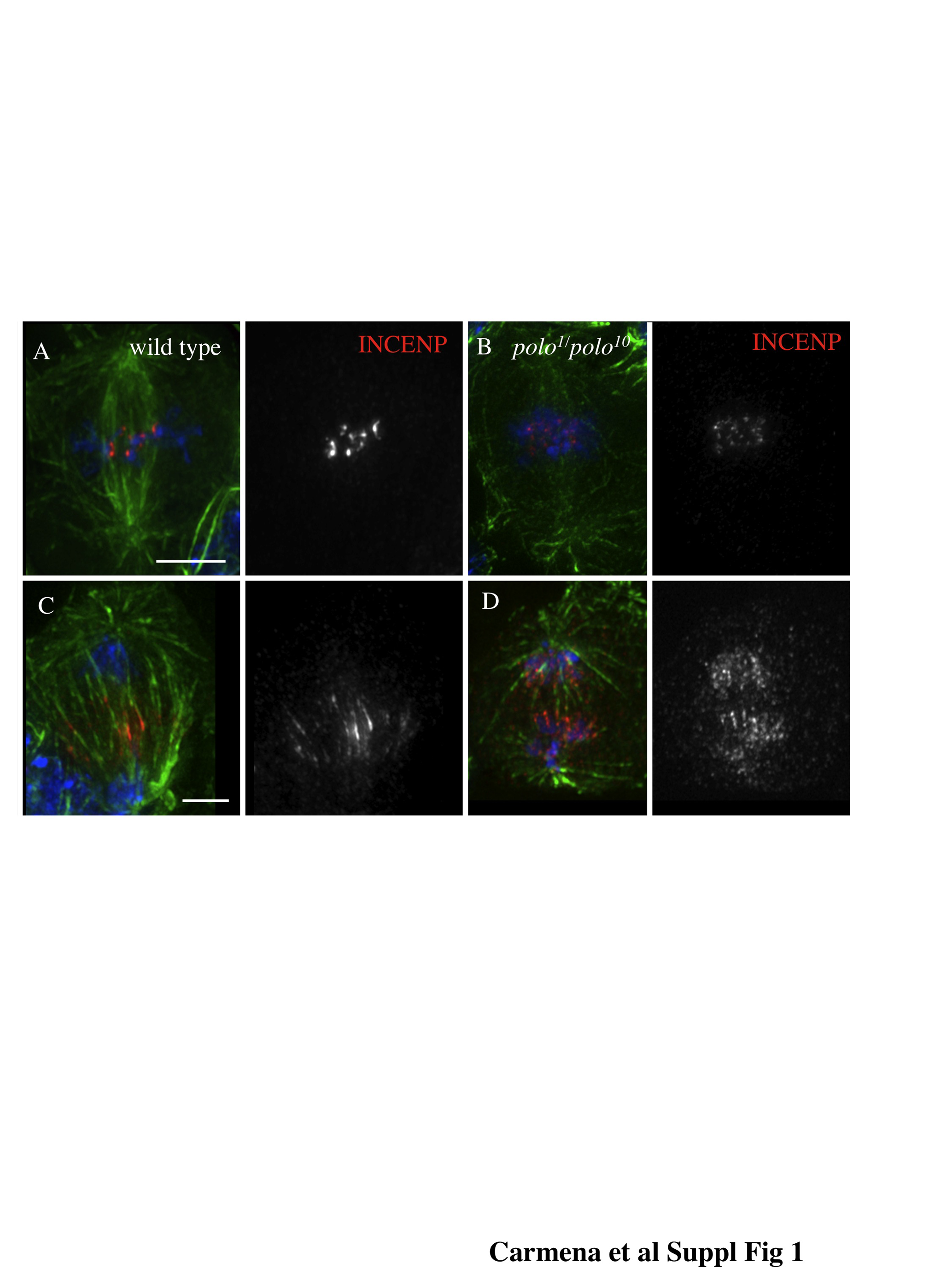

Supplement: Supplemental Figure 1 [file rsob140162supp2.tif]

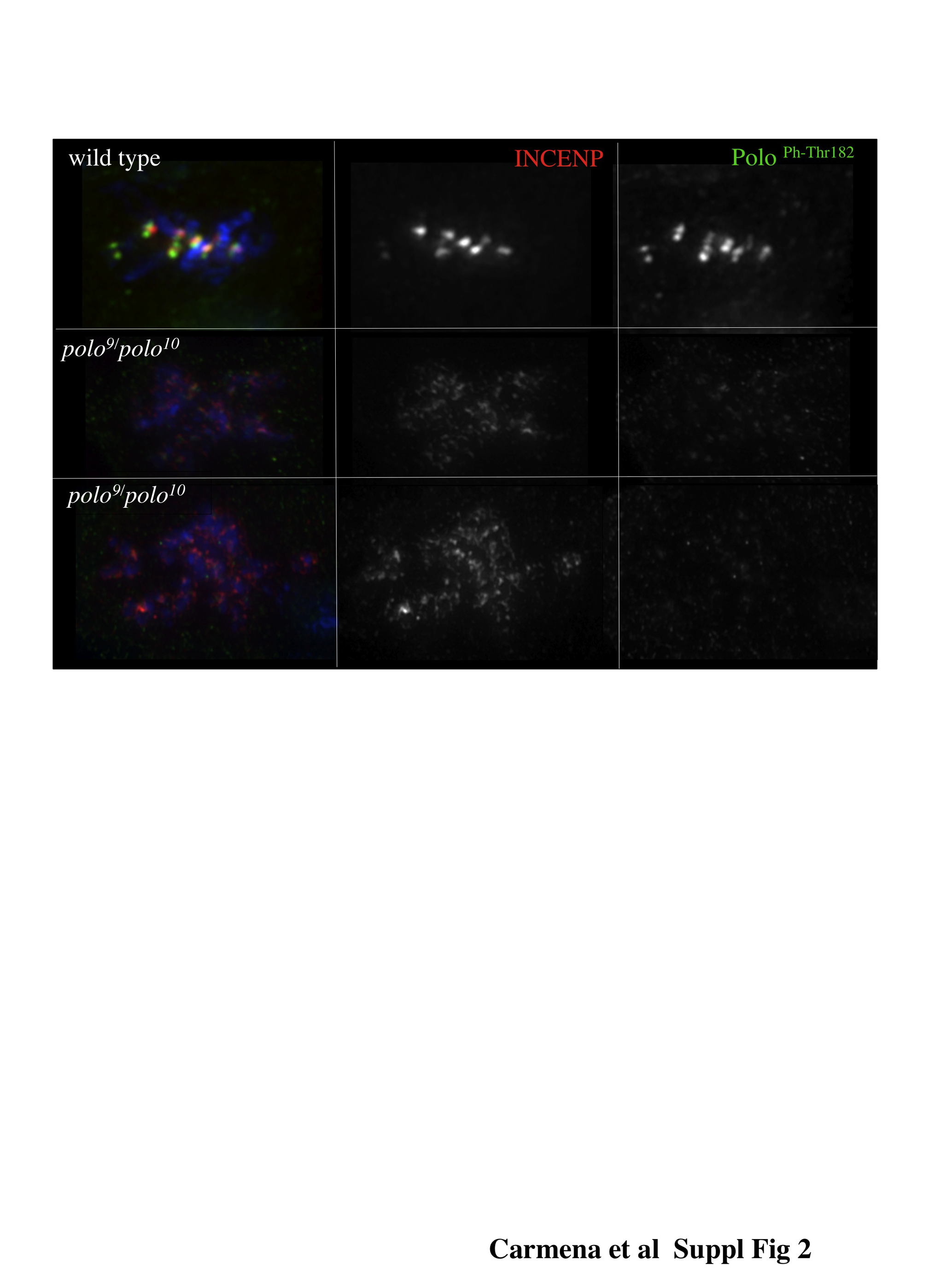

Supplement: Supplemental Figure 2 [file rsob140162supp3.tif]

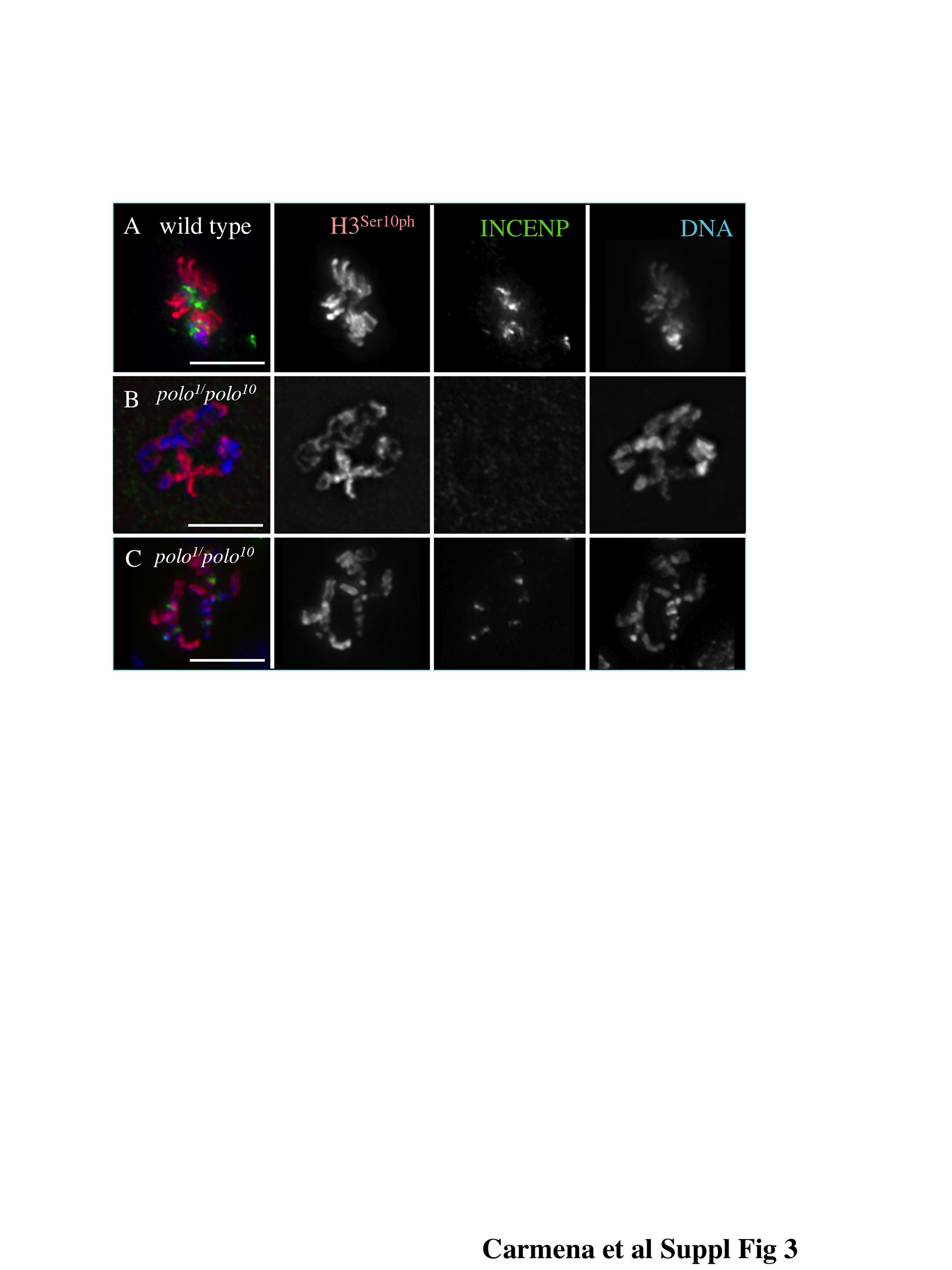

Supplement: Supplemental Figure 3 [file rsob140162supp4.tif]

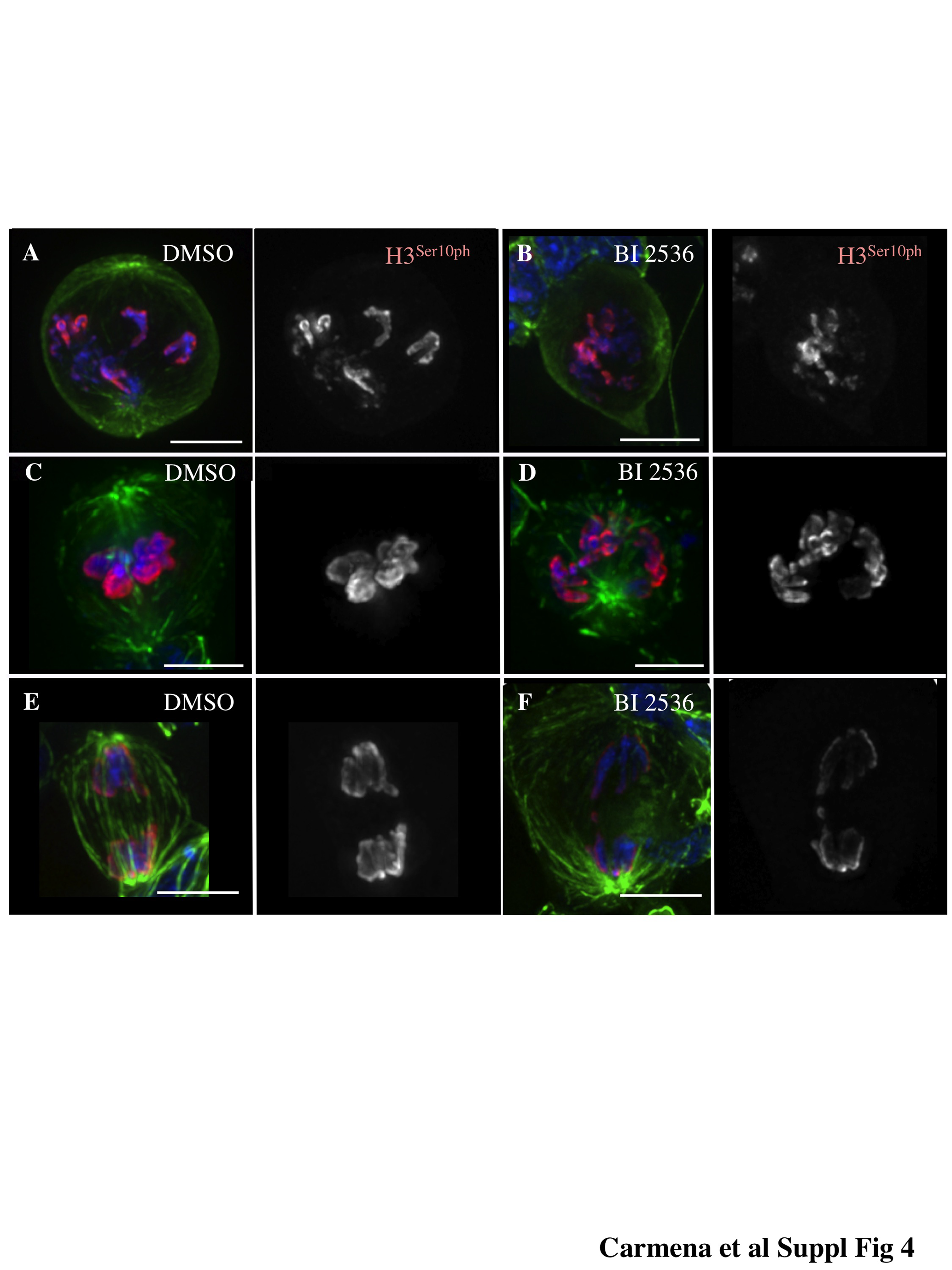

Supplement: Supplemental Figure 4 [file rsob140162supp5.tif]
